# Multidrug-resistant bacterial isolates from patients suspected of nosocomial infections at the University of Gondar Comprehensive Specialized Hospital, Northwest Ethiopia

**DOI:** 10.1186/s13104-018-3709-7

**Published:** 2018-08-20

**Authors:** Tigist Feleke, Setegn Eshetie, Mulat Dagnew, Mengistu Endris, Wondwossen Abebe, Moges Tiruneh, Feleke Moges

**Affiliations:** 1 Department of Hospital Laboratory, University of Gondar Comprehensive Specialized Hospital, P.O. Box: 196, Gondar, Ethiopia; 2 Department of Medical Microbiology, School of Biomedical and Laboratory Sciences, College of Medicine and Health Sciences, University of Gondar, Gondar, Ethiopia

**Keywords:** Nosocomial infections, Multidrug resistant, Gondar

## Abstract

**Objectives:**

As the hospital environment favors the circulation of drug resistant bacteria, continuous surveillance of antibiotic resistant patterns is an important approach for a better patient management. This study is therefore, aimed to assess multidrug resistant bacterial isolates from patients suspected of nosocomial infections at the University of Gondar Comprehensive Specialized Hospital, Gondar, Ethiopia.

**Results:**

Of the 260 patients, 173 (66.5%) of them were culture positive. Among culture positive patients a total of 216 bacterial isolates were recovered, of which the most common species were *S. aureus* 77 (35.6%), followed by *E. coli* 33 (15.3%) and *Klebsiella* spp 29 (13.4%). Of the *S. aureus* isolates, 67.5% were cefoxitin (methicillin) resistant. C*itrobacter* spp (100%), *Klebsiella* spp (79.3%) and *E. coli* (75.3%) were the leading MDR Gram-negative isolates. The overall MDR resistant rate was 152 (70.4%).

## Introduction

The impact of nosocomial infections (NIs) is an important public health concern due to the presence of increasing numbers of immunocompromised hospitalized patients, emergence of new strains of antibiotic resistant pathogens [1, 2]. It is largely known that bacteria are the leading cause of NIs [3]. The most common types of NIs that could occur in a hospital set up are: surgical site infections, blood stream infections, urinary tract infections [4] respiratory infections, gastroenteritis, pneumonia and meningitis and other soft tissue infections [5].

Currently, antibiotics remain the leading therapy for treating bacterial infections. However, the irrational use of antibiotics is no longer viewed as benign and certain strains of multidrug resistant bacteria have emerged by selection pressure; as a result, bacteria that have been once sensitive, re-emerged as resistant to different antibiotics and create limited therapeutic options, increased risks of treatment failure and poor patient management. As the incidence of antibiotic resistance rises, so do costs associate with its consequences. The worldwide emergence of multidrug resistant (MDR) among Gram-negative and Gram-positive bacteria has resulted in a great threat to efforts against bacterial pathogens [6]. It is clear that if there is no effective timely response, the challenge of antibiotic resistance is becoming alarming and will be a great challenge in the years to come. The aim of this study is therefore, to assess the rate of multidrug resistant bacterial isolates from patients suspected of nosocomial infections at the University of Gondar Comprehensive Specialized Hospital, Northwest Ethiopia.

## Main text

### Methods

#### Study area, study period and study population

The study was conducted at the University of Gondar Comprehensive Specialized Hospital, Northwest of Ethiopia from February 1 to May 31, 2016. All inpatients in the hospital wards that were tentatively diagnosed by the responsible physicians who were suspected of nosocomial infection were study populations. Nosocomial infection is an infection occurring after 48 h of hospital admission or 3 days after discharge or infection occurs within 30 days after the operation [7].

#### Sample size and sampling technique

The sample size was determined by using a single population proportion formula using the prevalence rate of 16.4% [8] a total of 260 samples were included in the study. The study participants were enrolled consecutively using convenience sampling technique.

#### Data collection and laboratory methods

Data were collected using structured questionnaire consisting of the patient’s demographic information. Clinical samples such as blood (2 mL for children and 10 mL for adults), pus, wound discharge, morning mid-stream urine (3 mL), stool, eye discharge and body fluids were aseptically collected using sterile containers and transported promptly to the Microbiology laboratory with appropriate transport media. Identification of bacteria to species level was done using colony characteristics, gram reaction and different biochemical tests following standard procedure [9] and antimicrobial susceptibility testing of each isolates was checked using Muller-Hinton agar (Oxoid, England). Different antibiotic discs were used (Oxoid, England) following Kirby Bauer disc-diffusion method and interpreted following CLSI guidelines [10].

#### Data analysis and interpretation

Data were entered, and analyzed using SPSS version 23 software. Results were presented through graphs and tables. Statistical significance association was measured by using Chi square test and p value < 0.05 was considered as statistically significant.

### Results

A total of 260 hospitalized patients suspected of having nosocomial infections were assessed by culture. The 152/260 (58.5%) of the study subjects were females. The age of the patients ranged from 1 to 85 years with the median age of 27.61 ± 20.3 years. The clinical samples collected were mainly wound discharge, pus, urine, blood, stool, eye discharge and body fluids (Table 1).

**Table 1:** Socio-demographic characteristics of inpatients suspected for nosocomial bacterial infections at the University of Gondar Comprehensive Specialized Hospital, 1st February–31st May 2016

Characteristic	Culture positivity rate		
	Yes n = 173 (%)	No n = 87 (%)	Total n = 260 (%)
Age (years)			
≤ 15	44 (25.4)	36 (41.4)	80 (30.8)
16–30	63 (36.4)	20 (23)	83 (31.9)
≥ 31	66 (38.2)	31 (35.6)	97 (37.3)
Sex			
Male	67 (38.8)	41 (47.1)	108 (41.6)
Female	106 (61.3)	46 (52.9)	152 (58.5)
Occupation			
Civil servant	29 (16.8)	18 (20.7)	47 (18.1)
Farmer	41 (23.7)	19 (21.9)	60 (23.0)
Student	30 (17.3)	11 (12.6)	41 (15.8)
Private	46 (26.6)	14 (16.1)	60 (23.1)
Others	27 (15.6)	25 (28.7)	52 (20)
Educational status			
Illiterate	69 (39.9)	35 (40.2)	104 (40)
1st cycle complete	39 (22.5)	25 (28.8)	64 (24.6)
2nd cycle complete	36 (20.8)	10 (11.5)	46 (17.7)
Diploma and above	29 (16.8)	17 (19.5)	46 (17.7)
Marital status			
Single	77 (44.5)	47 (54)	124 (47.7)
Married	77 (44.5)	34 (39.1)	111 (42.7)
Divorced	19 (11.0)	6 (6.9)	25 (9.6)
Specimen			
Wound/pus	105 (60.7)	12 (13.8)	117 (45)
Urine	33 (19.1)	27 (31.1)	60 (23.1)
Blood	25 (14.5)	31 (35.6)	56 (21.5)
Stool	5 (2.9)	10 (11.5)	15 (5.8)
Eye discharge	4 (2.3)	1 (1.1)	5 (1.9)
Other body fluids^a^	1 (0.6)	6 (6.9)	7 (2.7)
Ward			
Surgical	68 (39.3)	14 (16.1)	82 (31.5)
Pediatrics	41 (23.7)	36 (41.4)	77 (29.6)
Medical	30 (17.3)	26 (29.9)	56 (21.5)
Gynecology	28 (16.2)	6 (6.9)	34 (13.1)
Mixed ward	6 (3.5)	5 (5.7)	11 (4.2)
Total	173 (66.5)	87 (33.5)	260 (100)

^a^ Other body fluids = brain abscesses, joint fluids, pleural fluids

Of the 260 patients’ specimens cultured, 173/260 (66.5%) of them were culture positive and 87/260 (33.5%) were culture negative. The highest prevalence of infection was found from Surgical ward 68/173 (39.3%) followed by Pediatrics ward 41/173 (23.7%), Medical ward 30/173 (17.3%) and Gynecology ward 28/173 (16.2%). Of the 173 clinical samples positive for culture, 216 bacterial isolates were identified (131 sample with single infection, 41 with double infection and 1 with triple infection). The most common clinical sample assessed was wound/pus 117/260 (45%), followed by urine 60/260 (23.1%) and blood 56/260 (21.5%) (Table 1). Out of 173 (66.5%) samples, a total of 216 bacterial isolates were recovered, of which 101/216 (46.8%) were Gram-positives and 115/216 (53.2%) were Gram-negatives. The most common isolates were *S. aureus* 77/216 (35.6%), *E. coli* 33/216 (15.3%) and *Klebsiella* spp 29/216 (13.4%).

Antibiotic resistance patterns of Gram positive bacteria showed that, *S. aureus* was resistant to penicillin 74/77 (96.1%), cefoxitin 52/77 (67.5%) (surrogate marker for methicillin-resistant *S. aureus* (MRSA)), amoxicillin 39/77 (50.6%), clindamycin 28/77 (36.4%) and cotrimoxazole 27/77 (35.1%). The resistance patterns of coagulase negative *Staphylococci* (CoNS) to penicillin were 14/14 (100%). Higher resistance rate to *S. pyogenes* was observed in ceftriaxone 6/10 (60%), amoxicillin and clindamycin 5/10 (50% each) and lowest resistance rate in penicillin 1/10 (10%) (Table 2).

**Table 2:** Antibiotic resistance patterns of Gram positive and Gram negative bacterial isolates at the University of Gondar Comprehensive Specialized Hospital, 1st February–31st May 2016

Isolate	ERY n (%)	FOX n (%)	PEN n (%)	AMX n (%)	AMX/C n (%)	TTC n (%)	CIP n (%)	F n (%)	CAF n (%)	CRO n (%)	DA n (%)	SXT n (%)	GEN n (%)	AM n (%)
*S. aureus* (n = 77)	17 (22.1)	52 (67.5)	74 (96.1)	39 (50.6)	18 (23.4)	16 (20.8)	16 (20.8)	9 (11.7)	13 (16.9)	11 (14.3)	28 (36.4)	27 (35.1)	11 (14.3)	NT
CoNS (n = 14)	7 (50)	NT	14 (100)	10 (71.4)	8 (57.1)	6 (42.9)	6 (42.9)	5 (35.7)	3 (21.4)	7 (50)	2 (14.3)	9 (64.3)	3 (21.4)	NT
*S. pyogenes* (n = 10)	3 (30)	NT	1 (10)	5 (50)	3 (30)	2 (20)	3 (30)	2 (20)	2 (20)	6 (60)	5 (50)	2 (20)	3 (30)	NT
*E. coli* (n = 33)	NT	NT	NT	21 (63.6)	18 (54.5)	23 (69.7)	13 (39.4)	15 (45.5)	9 (27.3)	14 (42.4)	NT	22 (66.7)	11 (33.3)	10 (30.3)
*Klebsiella* spp (n = 29)	NT	NT	NT	22 (75.9)	16 (55.2)	13 (44.8)	4 (13.8)	16 (55.2)	9 (31.0)	14 (48.3)	NT	14 (48.3)	11 (37.9)	5 (17.2)
*Citrobacter* spp (n = 15)	NT	NT	NT	13 (86.7)	10 (66.7)	12 (80)	4 (26.7)	9 (60)	4 (26.7)	7 (46.7)	NT	7 (46.7)	5 (33.3)	2 (13.3)
*E. aerogenes* (n = 10)	NT	NT	NT	9 (90)	4 (40)	2 (20)	0	5 (50)	2 (20)	4 (40)	NT	3 (30)	1 (10)	0
*Serratia* (n = 9)	NT	NT	NT	7 (77.8)	3 (33.3)	3 (33.3)	2 (22.2)	4 (44.4)	1 (11.1)	4 (44.4)	NT	2 (22.2)	1 (11.1)	1 (11.1)
Others^a^ (n = 19)	NT	NT	NT	11 (57.9)	8 (42.1)	12 (63.2)	3 (15.8)	14 (73.7)	3 (15.8)	6 (31.6)	NT	6 (31.6)	3 (15.8)	2 (10.5)

*CoNS  *coagulase negative *Staphylococci, AMX*  amoxicillin, *AMX/C*  amoxicillin-clavulanate, *CAF*  chloramphenicol, *CIP*  ciprofloxacin, *CRO*  ceftriaxone, *FOX*  cefoxitin, *DA*  clindamycin, *ERY*  erytromycin, *F*  nitrofurantoin, *GEN*  gentamycin, *P*  penicillin, *SXT*  cotrimoxazole, *TTC*  tetracycline, *AM*  amikacin, *NT*  not tested, ^a^ Others = (*Shigella* spp = 8*, Proteus* spp = 7*, Pseudomonas* = 3*, S. typhi* = 1)

Among Gram-negatives, *E. coli* was resistant to tetracycline 23/33 (69.7%), cotrimoxazole 22/33 (66.7%), amoxicillin 21/33 (63.6%), amoxicillin-clavulanate 18/33 (54.5%) and least resistant to chloramphenicol 9/33 (27.3%). *Klebsiella* spp were resistant to amoxicillin 22/29 (75.9%), amoxicillin/clavulanate and nitrofurantoin (each) 16/29 (55.2%) and cotrimoxazole and ceftriaxone (each), 14/29 (48.3%). *Citrobacter* spp were resistant to amoxicillin 13/15 (86.7%), tetracycline 12/15 (80%) and amoxicillin/clavulanate 10/15 (66.7%) *E. aerogenes* was 9/10 (90%) resistant to amoxicillin and 5/10 (50%) to nitrofurantoin. However, 90% of the isolates of *E. aerogenes* were susceptible to gentamicin and all isolates of *E. aerogenes* were susceptible to amikacin and ciprofloxacin (Table 2). Higher number of multi-drug resistant isolates were recorded in the present study in *Citrobacter* spp 15/15 (100%), followed by CoNS12/14 (85.7%), *Klebsiella* spp 23/29 (79.3%), *E. coli* 25/33 (75.8%), and *S. aureus* 47/77 (61%). The overall MDR resistant in this study was 152/216 (70.4%) (Table 3).

**Table 3:** Multidrug resistance patterns of bacterial isolates from inpatients suspected for nosocomial bacterial infections at the University of Gondar Comprehensive Specialized Hospital, 1st February–31st May 2016

Isolates	Degree of resistance									
	R0 n (%)	R1 n (%)	R2 n (%)	R3 n (%)	R4 n (%)	R5 n (%)	R6 n (%)	R7 n (%)	R ≥ 8 n (%)	MDR^a^ n = (%)
*S. aureus (*n = 77)	–	11 (14.3)	19 (24.7)	6 (7.8)	12 (15.6)	6 (7.8)	6 (7.8)	4 (5.2)	13 (16.9)	47 (61.0)
*E. coli* (n = 33)	–	5 (15.1)	3 (9.1)	4 (12.1)	2 (6.1)	4 (12.1)	1 (3.0)	1 (3.0)	13 (39.4)	25 (75.8)
*Klebsiella* spp (n = 29)	–	3 (10.3)	3 (10.3)	2 (6.9)	5 (17.2)	3 (10.3)	3 (10.3)	2 (6.9)	8 (27.6)	23 (79.3)
*Citrobacter* spp (n = 15)	–	–	–	4 (26.7)	3 (20)	1 (6.7)	1 (6.7)	1 (6.7)	5 (3.3)	15 (100)
CoNS (n = 14)	–	1 (7.1)	1 (7.1)	2 (14.3)	1 (7.1)	–	4 (28.6)	–	5 (36.7)	12 (85.7)
*E. aerogenes* (n = 10)		1 (10)	2 (20)	3 (30)	–	–	3 (30)	–	1 (10)	7 (70.0)
*S. pyogenes (*n = 10)	2 (20)	2 (20)	1 (10)	2 (20)	–	–	–	–	3 (30)	5 (50.0)
*Serratia* (n = 9)	–	1 (11.1)	3 (33.3)	1 (11.1)	1 (11.1)	1 (11.1)	–	1 (11.1)	1 (11.1)	5 (55.6)
Others^b^ (n = 19)	–	3 (1.6)	3 (1.6)	1 (5.3)	6 (31.6)		1 (5.3)	1 (5.3)	4 (21.1)	13 (68.4)
Total (n = 216)	2 (0.9)	27 (12.5)	35 (16.2)	25 (11.6)	30 13.9)	15 (6.9)	19 (8.8)	10 (4.6)	53 (24.5)	152 (70.4)

*CoNS* coagulase negative *Staphylococci*

^a^ MDR = isolates resistant to 3 or more antibiotics classes. R0 = all are sensitive, R1 = resistant to one antibiotic classes, R2 = resistant to two antibiotic classes etc., R ≥ 8 resistant to greater or equal to 8 antibiotics

^b^ Others *(Shigella* spp = 8*, Proteus* spp = 7*, Pseudomonas *= 3*, S. typhi *= 1)

### Discussion

The present study showed that 66.5% of the inpatients had bacterial infections. This result is in line with a study reported from Dessie, Northeast Ethiopia (70.5%) [11]. However, our result is lower than studies from Jimma Hospital (87.3%) and Addis Ababa Hospitals (84.1%) [12, 13]. These variations may be due to differences in clinical samples, methodological variations, and study settings. Of the 173 culture positive patients, 131 (75.7%) had single infections, 41 (23.7%) double infections and 1 (0.6%) triple bacterial infections. These findings were consistent with a report in Brazil, 23.3% with polymirobial infections [14]. However, slightly lower results of polymicrobial growth were reported by Sikka et al. [15], 14.3% and Singh et al. [16], 11.3%. The difference in polymicrobial infection might be due to the fact that, most of the patients were with surgical site infections, the patient-related factors, poor equipment quality, financial resources as well as the competence of the surgical team [17].

In the present study, high rate of Gram negative (53.2%) bacteria were the causative agents of nosocomial infections than Gram-positives (46.8%). Similarly, Gram-negative bacteria as the leading cause of nosocomial infection were reported from Addis Ababa Hospitals 76 (73%) [13] and Jimma Hospital 53% [12]. Furthermore, Gram-negative organisms constituted more than three quarters (78.3%) of all causative organisms in hospital associated infections in Ibadan, Nigeria than Gram positives [18]. The higher proportion of the Gram-negative bacteria might be ascribed that most study participants had under gone abdominal surgery and Gram negatives are predominantly reported tobe involved in intra-abdominal procedures [19].

*S. aureus* 77 (35.6%) and *E. coli* 33 (15.3%) were the most common isolates reported in this study. Similar results reported from Jimma, revealed that the predominant organisms isolated from wound infection were *S. aureus* 47 (32.4%) and *E. coli* 29 (20%) [12]. The most common clinical samples that revealed high frequency of isolation were wound/pus 136 (63%), followed by urine 40 (18.5%) and blood 29 (13.4%). These findings are in line with other studies done in Gabon and Ethiopia [17, 20]. On the contrary, the present study is different from other studies elsewhere [21–23]. The variations in this report may be due to host, microbial and environmental factors [24].

Among Gram-positive bacteria, *S. aureus* and CoNS were the most common isolates that causes nosocomial infections. This is in line with reports from India revealed that *S. aureus* and CoNS were commonly isolated Gram positive bacteria [25]. Of the total isolates of nosocomial infections S*. aureus* was the most common isolate, 35.6%. This was consistent from studies in Jimma, Nigeria and India [12, 26, 27]. S*. aureus* is a common pathogen due to its ubiquitous nature both as a normal skin flora and a pathogen in human host [28, 29]. Furthermore, MRSA isolation rate in this study was 67.5%. This was higher than 55% MRSA reported by Abera et al. [30] in Ethiopia. The high rate of prevalence in the present study may be due to the fact that like many other African countries MRSA prevalence is increasing from time to time [31].

Among Gram-negative isolates, E*. coli* (15.3%), *Klebsiella* spp (13.4%), *Citrobacter* spp (7.0%) were commonly isolated pathogens. This is consistent with culture results of different specimens in Iran which revealed that *E. coli* and *Klebsiella pneumoniae* were the most common isolates [32].

The antibiotic resistance patterns of the isolates were also assessed: hence high resistant *S. aureus* isolates were identified against penicillin (96.1%), amoxicillin (50.6%), clindamycin (36.4%) and cotrimoxazole (35.1%). Besides, of the *S. aureus* isolates, 67.5% were resistant to cefoxitin, a surrogate marker for MRSA [33]. Similarly, higher rate of MRSA (76.7%) was reported by Godebo et al. [34]. However, lower rate of MRSA reported by Nigussie et al. [35] and Latif et al. [36] as 38.5% and 31.25%, respectively. These variations may be due to the type of assessment used for diagnosis of MRSA and status of the hospital.

In this study *Citrobacter* spp (100%), *Klebsiella* spp (79.3%) and *E. coli* (75.3%) were the leading MDR Gram-negative isolates. The 100% MDR *Citrobacter* seen in this study is consistent with 100% MDR rate reported both in Jimma Hospital, Ethiopia and Pakistan [34, 37]. Overall multi-drug resistance rate in this study was 152 (70.4%). This result is consistent with results reported in South Africa [38] and Jimma Hospital (68.1%) [34] but relatively higher than reports from Dessie, 65.2% [10] and lower than from Bahir Dar Hospital 93.1% [39].

Based on the present study, 67.5% MRSA, 100% penicillin resistant CoNS, high rate of MDR in *Citrobacter* spp (100%), *Klebsiella* spp (79.3%) and *E. coli* (75.3%) were documented. The overall MDR rate was 70.4%. Therefore, finding high MDR isolates in this hospital warrants a need to establish a better diagnostics and functional antimicrobial stewardship.

## Limitation of the study

Addressing MRSA and MDR Gram negative bacterial pathogens from culture of 260 clinical samples may be timely. However, complete characterization of resistance mechanisms and clonal relatedness was not made and considered as a limitation of this study.

## Abbreviations

MDR: multidrug resistance (acquired resistance to three or more different classes of antimicrobial agents)
CoNS: coagulase negative *Staphylococci*
MRSA: methicillin resistant *S. aureus*
NIs: nosocomial infections
